# Emotion recognition and cognitive empathy deficits in adolescent offenders revealed by context-sensitive tasks

**DOI:** 10.3389/fnhum.2014.00850

**Published:** 2014-10-21

**Authors:** Maria Luz Gonzalez-Gadea, Eduar Herrera, Mario Parra, Pedro Gomez Mendez, Sandra Baez, Facundo Manes, Agustin Ibanez

**Affiliations:** ^1^Laboratory of Experimental Psychology and Neuroscience, Institute of Cognitive NeurologyBuenos Aires, Argentina; ^2^National Scientific and Technical Research CouncilBuenos Aires, Argentina; ^3^UDP-INECO Foundation Core on Neuroscience, Diego Portales UniversitySantiago, Chile; ^4^Universidad Autonoma del CaribeBarranquilla, Colombia; ^5^Human Cognitive Neuroscience, Psychology Department, University of EdinburghEdinburgh, UK; ^6^Scottish Dementia Clinical Research NetworkPerth, UK; ^7^Neuropsy and Biomedical Unit, Health School, University SurcolombianaNeiva, Colombia; ^8^Universidad del NorteBarranquilla, Colombia; ^9^Centre of Excellence in Cognition and its Disorders, Australian Research CouncilSydney, NSW, Australia

**Keywords:** offenders, adolescence, delinquency, social cognition, contextual processing, ecological tasks

## Abstract

Emotion recognition and empathy abilities require the integration of contextual information in real-life scenarios. Previous reports have explored these domains in adolescent offenders (AOs) but have not used tasks that replicate everyday situations. In this study we included ecological measures with different levels of contextual dependence to evaluate emotion recognition and empathy in AOs relative to non-offenders, controlling for the effect of demographic variables. We also explored the influence of fluid intelligence (FI) and executive functions (EFs) in the prediction of relevant deficits in these domains. Our results showed that AOs exhibit deficits in context-sensitive measures of emotion recognition and cognitive empathy. Difficulties in these tasks were neither explained by demographic variables nor predicted by FI or EFs. However, performance on measures that included simpler stimuli or could be solved by explicit knowledge was either only partially affected by demographic variables or preserved in AOs. These findings indicate that AOs show contextual social-cognition impairments which are relatively independent of basic cognitive functioning and demographic variables.

## INTRODUCTION

Adolescent offenders (AOs) are known to present with difficulties in emotion recognition and empathy. However, the nature of such affective impairments is still a matter of debate. While both emotion recognition and empathy require implicit integration of contextual social cues in complex environments, most tasks used to assess these domains in AOs can be solved through the application of abstract rules and explicit knowledge of social norms. In addition, performance on these tasks is thought to partially reflect the influence of basic cognitive skills, such as intellectual ability or executive functions (EFs). We propose that a more realistic approach to explore these difficulties may be afforded by context-sensitive and ecologically valid measures. In the present study, we investigated emotion recognition and empathy in AOs and non-offenders using tasks with different levels of contextual dependence and involvement of real-life scenarios. We also explored the impact of fluid intelligence (FI) and EFs on emotion recognition and empathy difficulties.

Facial emotion recognition is a context-sensitive process influenced by visual information, voices, bodies, and even words (Barrett et al., 2011; Ibanez et al., 2014b). Such a process is impaired in AOs (McCown et al., 1986; Jones et al., 2007; Marsh and Blair, 2008; Fairchild et al., 2009; Sato et al., 2009), as shown through tasks using static facial stimuli with a limited range of emotional expressions. Evidence from adult offenders suggests that difficulties in these tasks may result from confounding factors, such as low education or poor verbal IQ (Glass and Newman, 2006; Pham and Philippot, 2010; Domes et al., 2013).

AOs also exhibit impairments of empathy (Ellis, 1982; Burke, 2001; Lindsey et al., 2001; Jolliffe and Farrington, 2004; Decety et al., 2009; de Wied et al., 2012; Domes et al., 2013). This complex, context-sensitive domain (Melloni et al., 2014) refers to the capacity to share and understand the subjective experience of others in reference to oneself (Decety et al., 2012b). It involves both affective (sharing and responding to the emotional experience of others) and cognitive (understanding the intentions and perspectives of others) components. Some studies with both young and adult offenders have reported greater difficulties in cognitive than in affective empathy (Jolliffe and Farrington, 2004). However, more recent reports of adolescents and adults with marked psychopathic/antisocial traits (Jones et al., 2010; Schwenck et al., 2012; Lockwood et al., 2013a; Pasalich et al., 2014) revealed the opposite profile (i.e., more deficits in affective than cognitive components of empathy). Despite the complexity of empathy deficits in AOs, traditional studies have examined the issue using only self-report questionnaires, yielding mixed results. While some studies found significant differences between AOs and non-offenders (Ellis, 1982; Burke, 2001; Lindsey et al., 2001), others reported similar results for both groups (Kaplan and Arbuthnot, 1985; Lee and Prentice, 1988; Bush et al., 2000; Domes et al., 2013). Finally, studies in adult offenders found that the relationship between low empathy and offending behavior seems to be affected by IQ (Jolliffe and Farrington, 2004) and education (Domes et al., 2013). Empathy failures among adults and young offenders may also reflect executive dysfunction (Jolliffe and Farrington, 2004), although this proposal has not been tested heretofore.

Taken together, these studies suggest that factors such as IQ, education, and underlying cognitive functions may account both for deficits in emotion recognition and empathy in AOs and for the inconsistencies found in the literature. In several reports, AOs’ low verbal IQ was shown to tamper their general ability to solve cognitive tasks (Isen, 2010; Frisell et al., 2012) and was associated with low education level (Isen, 2010; Mottus et al., 2012). Likewise, there is evidence for the role of low IQ in EF deficits (Jolliffe and Farrington, 2004; Parra et al., 2005; Koolhof et al., 2007; Kennedy et al., 2011; Frisell et al., 2012).

Whereas verbal IQ depends on previous knowledge, FI reflects an individual’s capacity for abstract thought and reasoning (Catell, 1971). FI modulates affective and social cognition (Ibanez et al., 2013, 2014b). Although no previous study has assessed FI abilities in AOs, a recent report (Huepe et al., 2011) has found an association between low FI and poor psychosocial adaptation in adolescents under vulnerability conditions. However, to our knowledge, no previous study has investigated the differential contribution of FI and EFs to emotion recognition and empathy in AOs.

An important step to properly explore such issues is to acknowledge that both emotion recognition and empathy abilities require the integration of contextual cues in real-life scenarios (Ibáñez and Manes, 2012; Kennedy and Adolphs, 2012; Melloni et al., 2014). Nevertheless, most tasks used to assess these domains in AOs can be solved with explicit knowledge and fail to emulate the emotions and behaviors presented in real-life social interactions. As an alternative, we propose that difficulties in emotion recognition and empathy may be better understood through the use of ecologically valid, context-sensitive tasks requiring implicit inference of contextual cues (Baez et al., 2012, 2013, 2014c; Baez and Ibanez, 2014). Indeed, these tasks have proven sensitive to impairments in everyday activities in psychiatric populations (Burgess et al., 1998; Torralva et al., 2009a; Ibáñez and Manes, 2012; Melloni et al., 2014). Given that AOs manifest disruptive behavior and severe difficulties in daily social interactions (Carswell et al., 2004; Jolliffe and Farrington, 2004; Pardini and Fite, 2010), we suggest that context-sensitive measures may provide a more realistic approach to identify emotion recognition and empathy profiles in AOs.

Furthermore, recent evidence (Glass and Newman, 2009; Baskin-Sommers et al., 2011, 2012) suggests that emotional processing deficits in psychopathic individuals are explained by difficulties in processing contextual information. Particularly, a recent study on this population (Brazil et al., 2013) reported difficulties in the processing of contextual cues when this had to be done in an effortful way, with intact automatic use of cue-related information. We propose that AOs may exhibit similar difficulties, leading to failures in context-sensitive tasks.

In the present study we explore the performance of AOs in emotion recognition and empathy tasks involving real-life scenarios and two levels of contextual dependence. To this aim, we included: (i) emotion recognition tasks with low Emotional Morphing Task (EMT) and high The Awareness of Social Inference Test (TASIT) and Dual Valence Association Task (DVAT) context processing requirements, and (ii) two measures of empathy, namely, a self-report questionnaire Interpersonal Reactivity Index (IRI) with low contextual dependence and an ecologically valid task with high contextual integration demands Empathy for Pain Task (EPT). We have previously shown the high and low contextual dependence and involvement of real-life scenarios of these paradigms (Baez et al., 2012, 2013, 2014c; Baez and Ibanez, 2014). Here, we compared performance on these tasks between AOs and non-offenders while controlling for the effect of demographic variables (education and age). We also explored whether FI and EFs could partially predict the deficits of AOs in these domains. We hypothesize that AOs will exhibit primary deficits in emotion recognition and empathy tasks with high context-processing requirements. Conversely, we predict that their performance in tasks with low context-sensitivity will be preserved or explained by demographic or cognitive variables.

## MATERIALS AND METHODS

### PARTICIPANTS

Forty-six male participants (30 AOs and 16 non-offenders) were enrolled in the present study. The AOs were recruited from a reform school of young male offenders in Barranquilla, Colombia. According to file records, most of the AOs were recidivist (74%) and had been incarcerated between 4 and 48 months following murder (35%) or robbery (65%) sentences. The AOs completed a structured admission interview, aimed to confirm that they did not fulfill criteria for a life-time diagnosis of psychiatric disorders and were not under pharmacological treatment during the assessment. Although most AOs had a history of drug and/or alcohol use, none was diagnosed with addiction or was treated for this reason.

Non-offenders were recruited from higher schools located in the same district of residence of AOs. Recruitment was authorized and assisted by the schools’ principals and teachers. Inclusion criteria for control participants were: (a) gender (male), (b) age (between 15 and 18 years old), (c) education level (less than 12 years of education), and (d) absence of history of psychiatric or neurological disorders. All the adolescents provided informed assent and a parent or guardian provided informed consent in agreement with the Helsinki declaration. The study was approved by the ethics committee of CARI Mental Hospital of Barranquilla, Colombia.

### INSTRUMENTS

#### Emotion recognition assessment

***Low context-sensitive measure: facial emotion recognition***. We assessed facial emotion recognition using the EMT, which consists of photos of facial expressions featuring six basic emotions (happiness, surprise, sadness, fear, anger, and disgust). These images, taken from the Pictures of Affect Series (Ekman and Friesen, 1976), were morphed for each prototype emotion and for a neutral state (Young et al., 1997). The shape and texture differences between a neutral image (0%) and a full emotion face (100%) were manipulated in increments of 5% (500 ms for each image). The 48 morphed facial stimuli were randomly presented on a computer screen. Participants were asked to press a button as soon as they recognized the facial expression and then to identify it from a forced-choice list of six options. The images remained visible until the participant responded. We measured the mean accuracy of overall emotion recognition (maximum one point) and the accuracy for each emotion category. This task has been previously validated (Young et al., 1997) and used with other neuropsychiatric populations (Baez et al., 2012, 2013, 2014c; Couto et al., 2013; Cardona et al., 2014).

***High context-sensitive measure: contextual inference of emotional states***. We assessed contextual inference of emotional states through The Awareness of Social Inference Test (TASIT). The TASIT is a validated clinical test of social perception (McDonald et al., 2006) and has been extensively used to evaluate contextual inference of emotions in different neuropsychiatric disorders (Rankin et al., 2009; Baez et al., 2012, 2013, 2014c; Couto et al., 2013). This task requires time-efficient processing of contextual cues (e.g., prosody, facial movement, and gestures) taxing selective attention and social reasoning. Such demands are absent in tasks involving static stimuli. Specifically, we used part 1 of the TASIT – the emotion evaluation test (EET)–, which comprises 20 short (15–60 s-long) videotaped vignettes of trained professional actors interacting in everyday situations. After viewing each scene, participants must choose the emotion expressed by the focused actor (fear, surprise, sadness, anger, disgust) from a forced-choice list. A global score was calculated from the sum of accurately recognized trials (maximum 20 points) and for each emotional state (maximum four points for each one).

***High context-sensitive measure: emotional interference***. We also included a Dual Valence Association Task (DVAT) to measure emotion recognition under interference effects produced by a double categorization of valences and stimuli. This is a validated task (Ibanez et al., 2011) based on implicit association principles (Greenwald et al., 1998; Ibáñez, 2010). Participants must classify faces and words as either positive or negative by pressing a left or right key, respectively. The stimulus set includes pictures of happy and angry faces, and pleasant and unpleasant words. There are 10 stimuli per category. The task is organized in two blocks of 35 randomized trials in which words and faces are alternately presented for 300 and 100 ms, respectively. In congruent trials, stimuli must be categorized as angry-unpleasant (left) or happy-pleasant (right). In incongruent trials, the words must be categorized in the same manner while face categories appear on the opposite side of the screen in angry-pleasant (left) or happy-unpleasant (right) configurations. Thus, the latter trials require participants to inhibit the implicit contextual association of both emotional valence categories (e.g., a happy face with a pleasant word). A DVAT score was calculated for each subject based by subtracting mean reaction times of congruent blocks from those of incongruent blocks. In addition, we calculated the mean accuracy of both congruent and incongruent blocks (maximum 35 points).

#### Empathy assessment

***Low context-sensitive instrument: self-report questionnaire***. We assessed empathy through the IRI, a validated self-report questionnaire (Davis, 1983) extensively used for research on AOs (Jolliffe and Farrington, 2004; Lovett and Sheffield, 2007). The IRI includes 28 items that separately measure the cognitive and affective components of empathy. The instrument contains four scales: perspective taking (PT), empathic Concern (EC), Fantasy (F), and personal distress (PD).

***High context-sensitive measure: ecological/behavioral task***. We also administered an adaptation of an EPT previously validated with behavioral measures, eye-tracking, and fMRI (Decety et al., 2012a). It has been used to evaluate empathy deficits in psychopathic offenders (Decety et al., 2013), adolescents with psychopathic traits (Marsh et al., 2013), adolescents with antecedents of social deprivation (Escobar et al., 2014), and children with conduct disorders (Lockwood et al., 2013b). Our adapted version has been used in the assessment of other neuropsychiatric populations (Baez et al., 2012, 2013, 2014a,c; Couto et al., 2013; Baez and Ibanez, 2014). This task assesses empathy in the context of intentional and accidental harm. The task consists of 25 animated scenarios (11 intentional, 11 accidental, 3 neutral) involving two individuals. Each scenario consists of three digital color pictures presented in succession to imply motion. Three types of situations are depicted: (i) intentional harm, in which one person is in a painful situation intentionally caused by another (e.g., purposely stepping on someone’s toe); (ii) accidental harm, where one person is in a painful situation accidentally caused by another; and (iii) control or neutral situations (e.g., one person receiving a flower from another).

Since the protagonists’ faces were not visible, participants could not rely on them to guess emotional reactions. However, body expressions and postures provided sufficient information about the emotional reaction of the victim and the intention of the agent. Participants were asked to respond to three pair’s different questions. The first pair addressed cognitive aspects of empathy, namely (a) intentionality (Was the action done on purpose?) and (b) intention of the perpetrator to hurt the victim (How bad was the purpose?). The second pair tapped affective aspects, namely, (c) emphatic concern (How sad do you feel for the victim?), and (d) degree of discomfort (How upset do you feel for what happened in the situation?). The third pair assessed moral evaluation, namely (e) correctness of the action (How inappropriate was the action?), and (f) punishment (How much penalty does this action deserve?). The question about the intentionality of the action was answered by selecting Yes/No. The other questions were answered using a computer–based visual analog scale (ranging from -9 to 9), whose numbers were not visible to participants. The meaning of the scale’s extreme values depends on the question. For example, for the question “How sad do you feel for the hurt person?,” one extreme of the bar reads “I feel very sad” and the other one reads “I do not feel sad at all.” Performance was assessed considering the percentage of accuracy for the intentionality question and the ratings for the other questions.

#### Fluid intelligence

The Raven’s Standard Progressive Matrices (RSPM; Raven et al., 2008) was used as a measure of FI. Participants completed a series of drawings by considering the spatial organization of an array of objects, identifying relevant features, and choosing one object that matched one or more of the identified features.

#### Executive functions

We used the Institute of Cognitive Neurology (INECO) Frontal Screening test (IFS; Torralva et al., 2009b), which assesses frontal lobe function as indexed by the following subtasks: motor programming, conflicting instructions, Verbal Inhibitory Control, Abstraction Ability (proverbs interpretation), Backward Digit Span, Spatial Working Memory, and Go/NoGo. This task has been used with different neuropsychiatric populations (Torralva et al., 2009b, 2012; Fiorentino et al., 2013; Baez et al., 2014b) A mean total score is calculated from the sum of the subtask scores (30 points). A 25-point cutoff score has shown a sensitivity of 96.2% and a specificity of 91.5% in detecting patients with dysexecutive syndrome (Torralva et al., 2009b).

### DATA ANALYSIS

One-way repeated measures analysis of variance (ANOVA) and Tukey’s HSD *post hoc* tests were used (when appropriate) to analyze differences between groups in emotion recognition and empathy tasks. Demographic, FI, and EF data were compared between groups using student’s *t*-tests. To control for the effect of demographic and cognitive variables on our experiments, we first matched the groups in term of education and FI. Second, we used age and years of education as covariate variables in an ANCOVA. We reported the effects both before and after covariation. Finally, we conducted multiple regression analyses to explore whether emotion recognition and empathy deficits were partially explained by FI and EFs. The emotion recognition and empathy measures that were significantly different between groups after covariance analyses were separately considered as dependent variables. Group, RSPM score, and total IFS score were included as predictors. The α value for all statistical tests was set at 0.05. Eta squared (*n*^2^) was used as a measure of effect size for significant effects.

## RESULTS

### DEMOGRAPHIC DATA

**Table 1** shows no significant group differences in education [*t*_(44)_ = 0.821, *p* = 0.416]. Although both groups had very similar age, significant differences were found [*t*_(44)_ = -3.73, *p* = 0.001] and further ANCOVA was performed to control this variable.

**Table 1:** Demographic, neuropsychological and behavioral data.

	Adolescent offenders (*N* = 30)	Non-offenders (*N* = 16)	p*
Age	16.67 (0.54)	16 (0.63)	0.001
Education (years)	6.50 (1.77)	7.25 (1.88)	0.416
Fluid intelligence (RSPM)	48.53 (4.51)	49.44 (4.38)	0.517
**Executive functions (EFS)**
Motor programming	2.70 (0.46)	3 (0.00)	0.014
Conflicting instructions	2.96 (0.18)	2.75 (0.44)	0.024
Verbal inhibitory control	3.86 (1.79)	5.50 (0.73)	0.001
Abstraction (proverbs)	1.40 (0.89)	2.81 (0.40)	0.000
Backward digit span	2.83 (1.05)	2.25 (1.18)	0.094
Spatial working memory	3.06 (1.22)	3.93 (0.25)	0.008
Go/NoGo	2.83 (0.46)	2.93 (0.25)	0.407
IFS global score	20.86 (3.54)	24.93 (1.65)	0.000

* Student *t*-test. RSPM, Raven’s Standard Progressive Matrices; IFS, INECO Frontal Screening.

### GROUP DIFFERENCES IN EMOTION RECOGNITION AND EMPATHY

#### Emotion recognition

**FIGURE 1:**
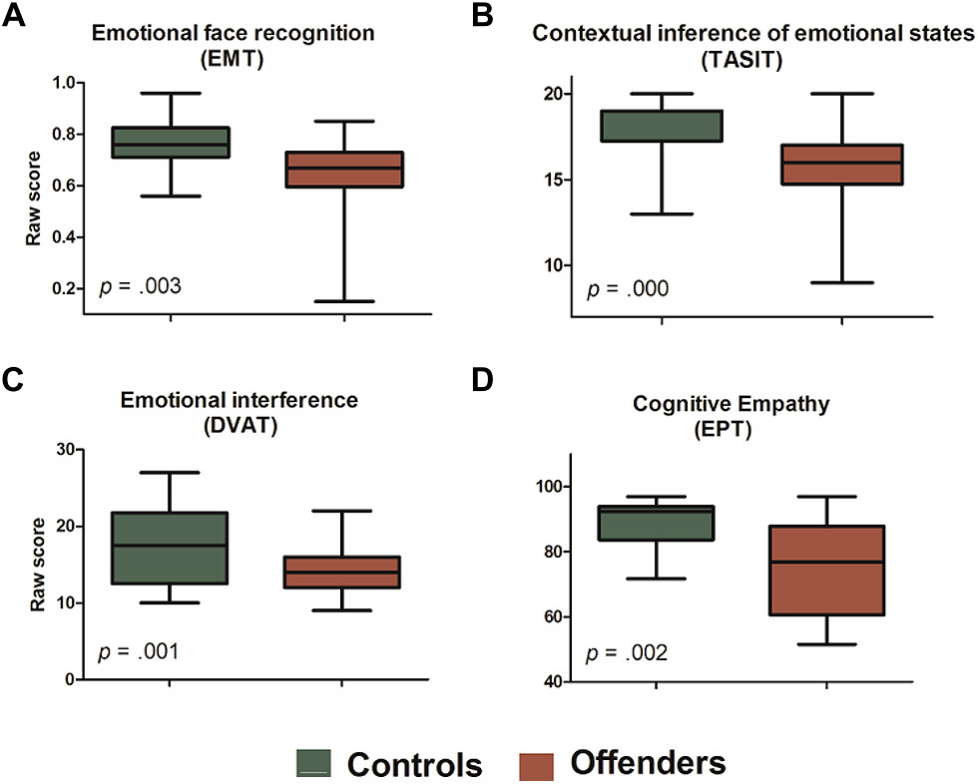
**Significant group differences in emotion recognition and empathy tasks. (A)** Emotional Morphing Task (EMT): mean accuracy and SD of emotion recognition. Note that group differences disappeared after adjusting for age (see Low Context-Sensitive Measure: Facial Emotion Recognition). **(B)** Awareness of Social Inference Test (TASIT): mean accuracy and SD for global score. **(C)** Dual Valence Association Task (DVAT): mean accuracy and SD for incongruent blocks. **(D)** Empathy for Pain Task (EPT): cognitive components (percentage and SD for intentionality question).

***Low context-sensitive measure: facial emotion recognition***. Relative to non-offenders, AOs had a significantly poorer performance on the EMT [*F*_(1,44)_ = 9.61, *p* = 0.003, *n*^2^ = 0.17; see **Figure 1A**]. This effect persisted after co-varying for education [*F*_(1,43)_ = 8.99, *p* = 0.004, *n*^2^ = 0.17] but became marginally significant after adjusting by age [*F*_(1,43)_ = 3.85, *p* = 0.056, *n*^2^ = 0.08]. A repeated-measures analysis including type of emotions revealed no interaction between emotions and groups [*F*_(5,220)_ = 1.00, *p* = 0.417; see details in **Table 2**].

**Table 2:** Means (SD) and group comparisons in the emotion recongnition and empathy task with low and high level of contextual dependence.

					Adolescent offenders	Non-offenders	p*
Emotion recognition	Low context-sensitive	Emotional Morphing Task (EMT)		Happiness Surprise Sadness Fear Anger	0.87 (0.23) 0.67 (0.27) 0.64 (0.25) 0.53 (0.25) 0.65 (0.24)	0.97 (0.05) 0.86 (0.15) 0.71 (0.22) 0.64 (0.21) 0.71 (0.17)	0.972 0.321 0.999 0.964 0.992
	
	High context-sensitive	The Awareness of Social Inference Test (TASIT)		Disgust Fear Surprised Sadness Anger Disgust	0.46 (0.31) 3.2 (0.88) 3.13 (1.19) 2.8 (0.8) 3.4 (0.85) 2.76 (0.89)	0.69 (0.18) 3.5 (0.63) 3.62 (0.61) 3.56 (0.63) 3.81 (0.4) 3.5 (0.63)	0.075 0.979 0.677 0.098 0.856 0.131
		
		Dual Valence Association Task (DVAT)		Congruent blocks Incongruent blocks DVAT global score	24.83 (0.63) 14.2 (0.73) 1.24 (3.37)	25.12 (0.86) 17.68 (1.01) 5.46 (7.23)	0.112 0.001 0.418

Empathy	Low context-sensitive	Interpersonal Reactivity Index (IRI)		Perspective taking Empathic concern Fantasy Personal distress Total score ERI	21.43 (3.47) 26.73 (3.88) 18.13 (84,21) 14.03 (4.31) 80.33 (9.12)	19.93 (3.47) 26.62 (4.46) 16.43 (2.78) 12.00 (3.26) 75.00 (7.26)	0.171 0.931 0.155 0.107 0.057
	
	High context- sensitive	Empathy for Pain Task (EPT)	Cognitive components	**Intentionality** Intentional Accidental Neutral	87.27 (11.09) 72.72 (15.47) 66.66 (38.15)	94.88 (7.39) 85.79 (9.44) 85.41 (70.45)	0.865 0.012 0.000
				
				**Intention to hurt** Intentional Accidental Neutral	6.02 (2.02) -2.74 (2.75) -6.40 (2.13)	5.46 (2.35) -1.76 (3.45) -5.08 (4.28)	0.985 0.858 0.640
				
			Affective components	**Empathic concern** Intentional Accidental Neutral	5.18 (2.70) 1.59 (3.89) -4.27 (3.06)	4.20 (2.83) 0.41 (3.34) -0.53 (3.39)	0.924 0.345 0.999
				
				**Discomfort** Intentional Accidental Neutral	5.14 (2.95) 1.03 (3.92) -5.01 (2.79)	4.99 (2.71) 0.41 (3.16) -5.22 (4.18)	0.999 0.990 0.999
				
			Moral components	**Correctness** Intentional Accidental Neutral	4.70 (3.17) -1.22 (3.41) -4.22 (3.21)	6.02 (1.96) -0.82 (3.49) -4.80 (4.04)	0.735 0.993 0.992

				
				**Punishment** Intentional Accidental Neutral	4.35 (2.87) -2.99 (3.03) -6.08 (2.03)	5.38 (2.44) -1.41 (3.66) -5.21 (4.23)	0.878 0.538 0.937

^*^ Tukey post-hoc analyses between groups for each category.

***High context-sensitive measure: contextual inference of emotional states***. Significant group differences were found in the TASIT global score [*F*_(1,44)_ = 14.92, *p* = 0.000, *n*^2^ = 0.25], AOs identifying fewer emotions than non-offenders (see **Figure 1B**). This effect persisted after controlling for co-variables [education: *F*_(1,43)_ = 18.98, *p* = 0.000, *n*^2^ = 0.30; and age: *F*_(1,43)_ = 10.35, *p* = 0.002, *n*^2^ = 0.19]. No interaction between groups and emotion categories were observed [*F*_(4,176)_ = 0.67, *p* = 0.610; see details in **Table 2**].

***High context-sensitive measure: emotional interference***. A repeated-measures analysis with accuracy scores from the DVAT in congruent and incongruent blocks revealed a significant interaction between Group and Block [*F*_(1,44)_ = 4.62, *p* = 0.036, *n*^2^ = 0.09]. A *post hoc* analysis (Tukey’s HSD, *MS* = 14.13, *df* = 85.06) showed that AOs made more errors in incongruent blocks than non-offenders (*p* < 0.001; see **Figure 1C**). These effects persisted after co-varying for education [*F*_(1,43)_ = 5.04, *p* = 0.030, *n*^2^ = 0.10] and age [*F*_(1,43)_ = 3.90, *p* = 0.034, *n*^2^ = 0.08]. No significant differences between groups were found for the DVAT main score [*F*_(1,44)_ = 0.66, *p* = 0.418; see details in **Table 2**].

In summary, AOs exhibited deficits in emotion recognition tasks. While difficulties in isolated emotion face recognition were partially mediated by age, deficits in contextual emotional inference as well as in emotional interference (both task requiring contextual integration of emotional information) were independent from co-variables.

#### Empathy

***Low context-sensitive measure: self-report questionnaire***. No significant differences between groups were found for IRI total score [*F*_(1,44)_ = 4.07, *p* = 0.059] or any of its four subscales separately (see **Table 2**).

***High context-sensitive measure: ecological/behavioral task***. Regarding the cognitive components of empathy, AOs presented significantly poorer comprehension of the intentionality of pain situations than non-offenders [*F*_(1,44)_ = 10.97, *p* = 0.002, *n*^2^ = 0.20; see **Figure 1D**]. This effect was maintained after adjusting for co-variables [education: *F*_(1,43)_ = 10.09, *p* = 0.003, *n*^2^ = 0.19; and age: *F*_(1,43)_ = 5.39, *p* = 0.020, *n*^2^ = 0.11]. However, no significant differences were observed in ratings of intention to hurt [*F*_(1,44)_ = 0.93, *p* = 0.338]. Regarding the affective components, no group differences were found in empathic concern [*F*_(1,44)_ = 1.45, *p* = 0.234], or in the degree of discomfort [*F*_(1,44)_ = 0.21, *p* = 0.649]. No significant differences were observed in terms of moral aspects of empathy, either in the correctness-of-action [*F*_(1,44)_ = 0.38, *p* = 0.554] or the punishment [*F*_(1,44)_ = 3.01, *p* = 0.089] ratings. Finally, no significant interactions were found between Group and Situation (intentional, accidental, and neutral) across the different components of empathy (see details in **Table 2**).

In sum, AOs showed impairments in the cognitive components of empathy, as assessed by the context-sensitive task. Specifically, they had difficulties to identify intentionality in situations in which someone suffers harm (EPT). No significant differences were observed in the self-report measure of empathy (IRI).

### INDIVIDUAL DIFFERENCES IN FI AND EFs

No significant group differences were found in FI [*t*_(44)_ = 0.653, *p* = 0.517; see **Table 1**]. However, relative to non-offenders, AOs showed significantly poorer performance on EFs [IFS global score: *t*_(44)_ = 4.33, *p* = 0.0001] and on most of the IFS sub-scales [motor programming: *t*_(44)_ = 2.56, *p* = 0.014; conflicting instructions: *t*_(44)_ = -2.33, *p* = 0.024; verbal inhibitory control: *t*_(44)_ = 3.47, *p* = 0.001; abstraction: *t*_(44)_ = 5.97, *p* = 0.0001; and spatial working memory *t*_(44)_ = 2.78, *p* = 0.008]. No significant differences were observed in the backward digit span [*t*_(44)_ = -1.71, *p* = 0.094] and Go/NoGo [*t*_(44)_ = 0.83, *p* = 0.407] subtasks.

### ARE EMOTION RECOGNITION AND EMPATHY DEFICITS PARTIALLY EXPLAINED BY FI OR EFs?

**FIGURE 2:**
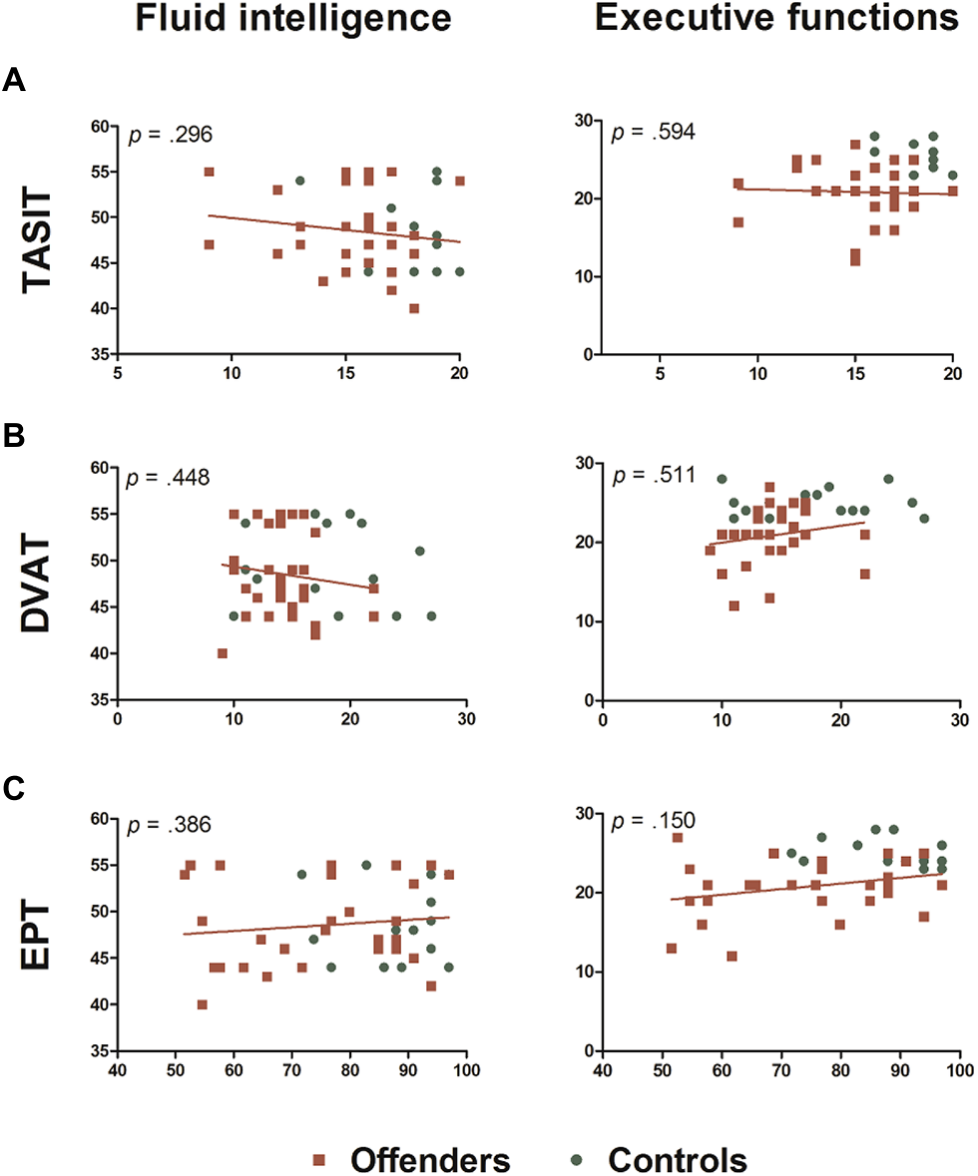
**Multiple regression analyses. (A)** Regression analysis using global score from TASIT as the dependent variable. **(B)** Regression analysis using the mean accuracy in incongruent blocks from the DVAT as the dependent variable. **(C)** Regression analysis using as dependent variable the mean percentage of correct responses in intentional, accidental, and neutral scenarios of cognitive aspect (intentionality) of the EPT.

Multiple regression analyses were performed to evaluate the influence of FI and EFs on the emotion recognition and empathy impairments observed in Group Differences in Emotion Recognition and Empathy section. **Figure 2** shows that none of the dependent variables were predicted by FI or EFs (see more results in **Table 3**).

**Table 3:** Coefficients of the multiple regression models of results reported in “Are Emotion Recognition and Empathy Deficits Partially Explained by FI or EFs?” section.

	Contextual inference of emotional states (TASIT)		Emotional Interference (DVAT)		Cognitive empathy (EPT)	
	β	*p*	β	*p*	β	*p*
Fluid intelligence (RSPM)	-0.14	0.296	-0.11	0.448	-0.12	0.386
Executive Functions (IFS)	-0.08	0.524	-0.11	0.511	-0.23	0.150
Group	-0.56	0.001	-0.34	0.052	-0.31	0.062

*TASIT, Awareness of Social Inference Test; DVAT, Dual Valence Association Task; EPT, empathy for Pain Task; RSPM, Raven’s Standard Progressive Matrices; IFS, INECO Frontal Screening.*

## DISCUSSION

In this study we employed a range of context-sensitive ecological measures to examine the emotion recognition and empathy profiles of AOs. Crucially, we controlled for the influence of demographic variables, such as age and education, and investigated the influence of FI and EFs on task performance. Our results showed that difficulties in tasks requiring contextual appraisal (TASIT, DVAT, and EPT) were not explained by covariates. However, performance on measures that included more simple stimuli or could be solved by explicit knowledge was either partially affected by demographic variables (EMT) or preserved in AOs (IRI). This study provides preliminary evidence that AOs exhibit social contextual processing impairments which are relatively independent from education, FI, or EFs.

### EMOTION RECOGNITION AND EMPATHY DEFICITS IN AOs

Previous studies have shown impairments in affective processing in AOs, specifically in the recognition of negative emotions (such as anger, disgust, fear, or sadness) during facial recognition tasks (McCown et al., 1986; Jones et al., 2007; Sato et al., 2009). In our study, we explored this domain through the EMT which includes a dynamic method for the presentation of facial expressions. The results thus obtained showed that AOs have a general difficulty in emotion recognition in the EMT, regardless of emotion type. However, these differences become marginally significant after adjusting for age. A previous study (Pham and Philippot, 2010) using a similar EMT with adult offenders found that deficits in decoding emotional facial expression were accounted for by education. In our study, AOs and non-offenders were matched by education, but group differences were found in age. Several studies (Thomas et al., 2007; Mancini et al., 2013) have reported that face emotion recognition abilities develop with age, with adults displaying more sensitivity to subtle changes in emotional expression than adolescents (Thomas et al., 2007). Therefore, differences in age affected performance on the EMT, possibly due to general effects of neurocognitive development. Taken together, previous and present results suggest that demographic variables, such as education and age, should be controlled for in order to unveil difficulties in basic emotion processing in AOs.

Moreover, emotion recognition difficulties in AOs were revealed by the TASIT, which requires the integration of cues from face, prosody, gesture, and social context to identify emotions (Baez et al., 2012, 2013; Baez and Ibanez, 2014). The age variable partially affected facial emotion recognition deficits, but it had no effect on the difficulties to infer more complex affective states. A previous study (Kipps et al., 2009) has demonstrated that contextual cues in the TASIT normally lead to more accurate emotion identification in healthy individuals. We suggest that performance on the TASIT may depend more on contextual integration skills than in basic facial emotion recognition abilities, which are associated with developmental trajectories (Thomas et al., 2007; Mancini et al., 2013).

We also found emotional interference difficulties in AOs assessed by the DVAT. Based on implicit association principles (Greenwald et al., 1998), this task assesses the interference effect produced by the implicit contextual association of categories with incongruent emotional valence (e.g., an angry face with a pleasant word). The performance on the DVAT requires the integration of emotional valence from facial expressions and semantic information in a highly associative context (Ibanez et al., 2011, 2014a). Our results showed that AOs were impaired at automatically discriminating two contextual opposed valences. This could be triggered by basic impairments of emotional binding processes, inhibitory control to deal with the interference effects in incongruent blocks, or a combination of both factors (Ibanez et al., 2011). We support the view that the AOs’ difficulties revealed by the DVAT and TASIT could be explained by a general impairment in integration of emotion and context.

Finally, to evaluate empathy we included both an ecological behavioral measure of empathy (the EPT) and a self-report questionnaire (the IRI). AOs showed deficits in the cognitive components of the EPT, but demonstrated no difficulties in the IRI. These behavioral results suggest affective-processing difficulties in AOs which are not revealed by self-report questionnaires. The IRI considers empathy as a trait and fails to fully represent empathic abilities because of its limited ecological validity (Ickes, 2009). Furthermore, AOs showed deficits in the cognitive components of empathy assessed by the EPT – specifically, in the comprehension of deliberately harmful actions. These deficits remained after covarying for demographic variables and were not predicted by FI or EFs.

It is important to note that the attribution of the action’s intentionality in the EPT is the main goal of the task and it is crucial to respond correctly to the affective and moral aspects associated with the actions observed in the task (Baez et al., 2013, 2014b). No difficulties were observed in the affective aspects of empathy. This result is consistent with previous findings suggesting that offending is more strongly associated with low cognitive empathy than low affective empathy (Jolliffe and Farrington, 2004). In addition, a recent study (Pasalich et al., 2014) found that callous-unemotional traits in adolescents with conduct problems were uniquely associated with deficits in cognitive empathy. Note, however, that most studies on adolescents with psychopathy or conduct disorders have reported greater deficits in the affective than in the cognitive aspects of empathy (Jones et al., 2010; Schwenck et al., 2012; Lockwood et al., 2013a). We suggest that both empathic processes can be difficult to separate, since the understanding of a cognitive aspect usually implies affective processing, and vice versa. This overlap between cognitive and affective components may partially explain the inconsistencies found in the literature and should be addressed by future studies.

In addition, the EPT results revealed that AOs had no difficulties to judge the correctness of actions performed by the perpetrator or the punishment deserved. This finding is consistent with previous evidence for adequate moral judgment in offenders (Cima et al., 2010; Schmoll, 2012; Radke et al., 2013).

In summary, we confirmed our prediction that emotion recognition and empathy deficits in AOs were described by tasks involving real-life scenarios and/or implicit contextual information (TASIT, DVAT, and EPT). These deficits were neither explained by demographic variables nor predicted by cognitive functioning. However, performance on the facial emotion recognition task (EMT), which has lower context-processing demands, was affected by age. In the same vein, AOs gave no signs of empathy deficits in the IRI questionnaire, probably due to the involvement of more explicit knowledge of social norms which would be preserved in these adolescents (Cima et al., 2010; Radke et al., 2013).

### DEFICITS IN PROCESSING CONTEXTUAL INFORMATION IN AOs

The present results suggest that AOs have difficulties in integrating affective processes with contextual information in ecological tasks. Although to our knowledge no previous study has addressed offender’s deficits to process contextual information, recent studies in psychopaths have begun to explore these difficulties within this population (Newman et al., 2010; Baskin-Sommers et al., 2013; Sadeh et al., 2013). These studies found that emotional deficits in psychopaths are moderated by difficulties to focus attention in complex scenarios. Thus, these authors proposed that information processing deficits in psychopathy could be explained by the interplay between attentional and emotional systems.

In the present study we found EFs impairments in AOs. The task used to assess such functions is known to tax top-down attentional function integrated in the frontal lobe. Although we failed to find associations between such tasks and measures of contextual processing in the assessed sample, such an association may still exist. For example, the ecological nature of the tasks used to assess contextual-dependent emotional and empathy processing may pose different demands on attentional functions that might denote other cognitive processes in these individuals. Future studies in AOs should explore whether deficits in ecological tasks that require contextual integration could be explained by attentional deficits which are also measured with more ecologically valid procedures. For example, studies with psychopathic individuals should consider exploring difficulties in contextual information processing in social cognition by using similar context-sensitive measures.

### LIMITATIONS AND FUTURE DIRECTIONS

Some important limitations of this study should be acknowledged. First, we did not include a measure of verbal IQ to control group differences between AOs and non-offenders. Low verbal IQ has been proposed as a confounding variable that may explain deficits in emotion recognition and empathy in adult offenders (Jolliffe and Farrington, 2004; Domes et al., 2013). However, we controlled the influence of education by selecting AOs and non-offenders with similar education level and including this variable as a covariate. Recent reviews suggest that low verbal IQ may be a consequence of adolescents’ truancy or verbal-educational deficits accumulated throughout childhood (Isen, 2010; Mottus et al., 2012). In other words, controlling education levels may indirectly control the effect of verbal IQ. Nevertheless, futures studies should consider including verbal IQ as a control measure of offenders’ cognitive task performance.

Second, we used the IFS as a screening measure of EFs. This test has been employed in several neuropsychiatric populations (Torralva et al., 2009b; Gleichgerrcht et al., 2011; Fiorentino et al., 2013; Baez et al., 2014b). However, AOs are known to exhibit deficits in inhibitory control and working memory (Bergvall et al., 2001; Koolhof et al., 2007; Verona et al., 2012). It would be desirable for future examinations to clarify the influence of these domains on emotion recognition and empathy tasks.

Third, since psychopathic/callous-unemotional traits seem to capture meaningful heterogeneity in AOs at the behavioral and neural levels (Sebastian et al., 2012; Viding et al., 2012; Lockwood et al., 2013b), they may be related to the observed emotion recognition and empathy deficits. Future studies should assess and control for the impact of these traits on emotional and empathy deficits in AOs.

Fourth, some methodological issues must be contemplated in future studies. We used several statistical tests to compare groups in tasks with different levels of contextual dependence. More sophisticated methods should be used in future research, including a parametric classification of low and high context-sensitive measures into a single general linear model.

Finally, our sample size was relatively small and only included male AOs. However, our sample was arguably large enough for the type of analyses performed (Hair et al., 2010), and it was not smaller than those in previous studies with offenders (Sato et al., 2009; Domes et al., 2013). Further research should assess the effect of context processing in emotion recognition and empathy domains in larger samples of AOs and extend these results in female population.

## CONCLUSION

Our study documents emotion recognition and empathy impairments in AOs. These are reflected by difficulties to integrate affective process (emotion and empathy) with contextual information in tasks that incorporate real-life scenarios. The results showed that AOs exhibit deficits in ecological, context-sensitive measures of emotion recognition and empathy (TASIT, DVAT, and EPT). Difficulties in these tasks were neither explained by demographic variables nor predicted by FI nor EFs. However, deficits in more basic emotion recognition tasks (EPT) were accounted for by age, while no difficulties were observed in measures that can be solved through explicit knowledge (IRI).

These results suggest that ecological measures are sensitive tools that should be applied in the assessment of AOs. Although implementation would be challenging, rehabilitation programs could aid AOs in the use of implicit rules to interpret contextual cues in real-life social environments.

## Conflict of Interest Statement

The authors declare that the research was conducted in the absence of any commercial or financial relationships that could be construed as a potential conflict of interest.
